# Progress in State Medicine

**Published:** 1903-08-22

**Authors:** 


*(To be Continued.)*

### Progress in State Medicine.

### MILK.

Marston[1] states that those actively engaged in the farming and dairy business are more closely associated with bacteria and their activities than any other class of persons. Modern dairying, apart from the method of keeping the cow, consists largely in trying to prevent bacteria from growing in milk, or in stimulating their growth in cream, butter, and cheese. Milk when secreted from the udder of a healthy cow contains no bacteria, and will undergo no change beyond a few physical changes connected with evaporation, and a slight oxidation of the fat, if kept free from them. This is almost impossible in ordinary dairying, and by the time the milk reaches the pail it may contain from half a million to a million bacteria per cubic inch of milk. Rapid growth of these organisms takes place in the milk left in the milk ducts from the last milking, and these are washed into the pail with the milk subsequently drawn. The hind quarters of cows usually contain much filth as the farmer rarely grooms them, and during milking, by the switching of their tails and by the rubbings from the milkers, no small amount of dirt is knocked off into the milk pail. Straining the milk, as usually practised, does not get rid of bacteria. The pails and cans, together with the dirty hands and clothes of the milkers are another source of bacterial infection. The air of the milking stall furnishes its quantum of bacteria. Market milk may contain in 24 hours as many as 500,000,000 of them per cubic inch. The souring of milk is due to the action of certain of the milk bacteria upon the milk sugar, which is converted into lactic acid, curdling the milk and giving it a sour taste. If the dairyman will keep his milk vessels clean, his cow as cleanly as his horse, attend to whitewashing, ventilation, air space, water supply, and drainage, he need never be disturbed by tainted milk. These precautions entail extra expense, and if adopted the public must expect to pay more for their milk. Cream as it is obtained from milk will always contain bacteria in large quantities, and they will grow in the cream as they will in the milk. Wilson,[2] referring to past legislation in regard to public health, points out that the Contagious Diseases (Animals) Act of 1878 was an acknowledgment that the conditions under which the milk supply of the country was carried out deserved some consideration. It was shown almost to the point of demonstration that milk had acted as the carrier of the infection of enteric fever, scarlet fever, and diphtheria. An Order of the Privy Council, known as the Dairies, Cowsheds, and Milk Shops Order, was issued in 1885, which provided that a local authority may make regulations for the inspection of cattle in dairies, for the prescribing and regulating the lighting, ventilation, cleansing, drainage, and water supply of dairies and cowsheds, for securing the cleanliness of milk-stores, milk-shops, and vessels used for containing milk for sale, for prescribing precautions to be taken by purveyors of milk and persons selling milk by retail against infection and contamination. Some of the smaller councils have been unwilling to adopt any regulations owing to the permissive nature of the powers. But every Council is definitely required by the Order to keep a register of persons who carry on the trade of cowkeeper, dairyman, or purveyor of milk, and such register is from time to time revised and corrected. A building is not to be occupied for the first time as a dairy or cowshed until reasonable requirements of the Council are satisfied as to the lighting, ventilation (including air space), cleansing, drainage, and water supply of the premises. He thinks the ideal manner in which cowsheds should be constructed is that the inner walls, to the height of 5 feet, should be formed of a smooth impervious material, so as to assist frequent cleansing. With regard to lighting the greatest amount possible should be obtained through the roof, supplementing windows in the walls by a number of glass tiles placed over every stall. He was disposed to think that too much stress had been laid upon securing proper air space to the exclusion of providing a definite standard of the methods which would procure adequate ventilation. There is a well-known objection on the part of the cowkeeper to alterations of his premises which would lower the temperature in the sheds, as this is followed by a lessened yield of milk. This result probably occurs mainly in those sheds where the cows are kept in an unnaturally warm, and also foul, atmosphere, and in sheds where cows are kept in buildings of adequate size and provided with sufficient ventilation, the result of a temporarily lessened temperature will be less noticeable so far as the quantity of milk can show. However, the object is to secure milk from healthy cows, and people had better be prepared to pay more for such a good supply rather than less for the poorer and rather larger quantity. He asks the question: Are our regulations in any degree responsible for the fact that during the year 1900 milk and cream to the value of £26,592 were imported into this country, and that £347,727 was paid for condensed milk imported in 1901. There is no security that the sources from which this immense quantity of milk was obtained were in a satisfactory condition. If the view be correct that the foreigner untrammelled by our regulations, is cutting out our farmers, and that the latter is unwilling to continue a trade in which he is troubled by inspection by outsiders, every endeavour must be made to bring it home to the farmer or cowkeeper as the seller, as well as the public as the purchaser, that all the regulations for every part of the milk trade are based upon the advice of many actually carrying on the trade; and that the lodging of animals under conditions conducive to their health and well-being are just the conditions with which the public and sanitary officers will be best satisfied. Attention[3] is drawn to the fact that freshly-drawn milk contains certain germicidal substances, which, when the milk is kept cool and free from gross contamination, will check the development of bacteria and even sometimes diminish their numbers. When the temperature of the milk is above 50° Fahr., or when the contamination is gross, the power of the germicidal substances is quickly exhausted. The whole of the milk drawn at a milking is not sterile, but the number of bacteria is small, and they are contained almost exclusively in the part of the milk first drawn. Experiments have shown that milk drawn with minute antiseptic precautions may be absolutely sterile after the udder has been half emptied. Park states that, in New York, milk purchased in ordinary shops averages in the coldest weather over 300,000 bacteria per cubic centimetre, in cool weather about 1,000,000, and in hot weather about 5,000,000. The milk may become infected during four stages: (1) Milking and treatment in the dairy; (2) transit to the city; (3) collection, manipulation, storage and distribution by the wholesale and retail dealers; (4) storage in the house of the purchaser. With regard to transit[4] the main precautions which the railway companies may fairly be asked to take in the interests of the public are three: (1) To supply covered sheds at the country stations where the milk churns may be kept under cover from the sun and dust when awaiting dispatch; (2) to supply well-ventilated vans in a systematic manner as a matter of routine; (3) to encourage the system of sending milk in sealed or locked churns. The large railway companies prefer to receive the churns locked, but the farmers can rarely be induced to adopt this system. Special[5] mention is made of the admirable precautions taken by four dairy companies for the safeguarding their milk from infection, two in London and two in Copenhagen.

[1] Sanit. Rec., March 12, 1903. [2] Ibid., March 19, 1903. [3] Brit. Med. Jour., March 21, 1903. [4] Ibid., April 4, 1903. [5] Ibid., April 11, 1903.

